# Supplementary material for: Drug repurposing for aging research using model organisms
Source: Aging Cell. 2017 Jun 16;16(5):1006–15. doi: 10.1111/acel.12626 (PMC5595691; doi:10.1111/acel.12626)
Supplement: Supplementary file 7 — Data S1 Zip‐Archive of all report cards. [file ACEL-16-1006-s007.zip › RC_P6G.pdf]

P6G

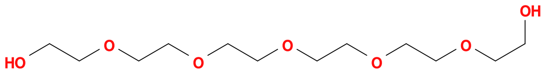

Database identifiers

ChEMBLCompound	CHEMBL1235082
CHEBI	49793
ZINC	ZINC04521548
eMolecules	492711

Ranking

	Rank	Score
Drosophila	426/697	0.336
C. elegans	380/591	0.153

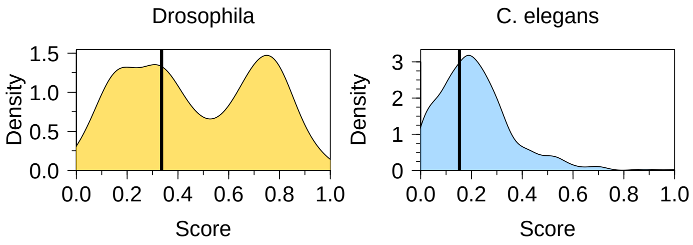

	Ageing implication	Domain conservation	Binding site conservation	Binding affinity	Bioavailability	Lipinski	Promiscuity	Purchasability	Drug approval	**Total**
Drosophila	1.0	0.97	0.798	0.352	(0.9)	0.0	-0.009	0.1	0.0	0.336
C. elegans	1.0	0.971	0.787	0.352	0.231	0.0	-0.009	0.1	0.0	0.153

Names

· **hexaethylene glycol**
· Hexagol
· hexaoxyethylene glycol
· PEG-6

Roles

ChEBI entry 49793 has no roles

Status

Approved drug (according to ChEMBL)	No
Number of Rule of 5 violations	0
Binding affinity to original target in log units (RF-Score prediction)	4.39
Burns *C. elegans* bioavailability prediction	-3.21

Compound Target Characteristics

Thioredoxin

Best gene implication in ageing for this target family came from gene Q9V429 annotated in UniProt release 2014_02. Annotation GO 8340 (determination of adult lifespan) was Inferred from Mutant Phenotype

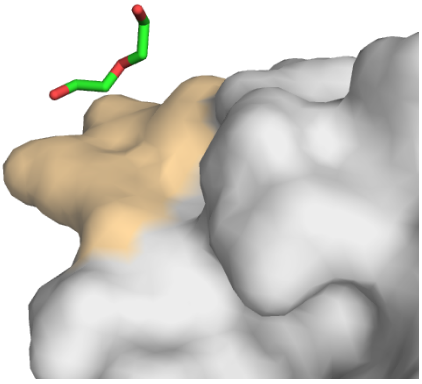

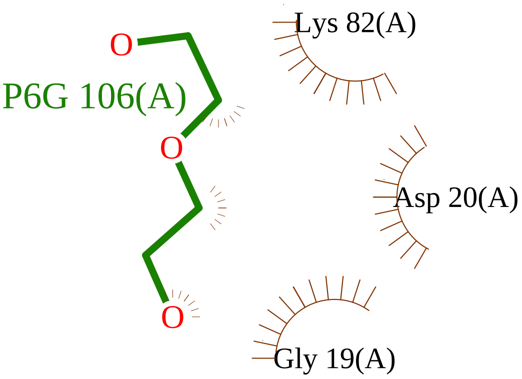

protein	amino acids contacts (binding site)
PDB:3e3e:chainA:P10599	G D K
tr:H9ZYJ2:H9ZYJ2_HUMAN	G D K
sp:P10599:THIO_HUMAN	G D K
tr:R4GNK3:R4GNK3_RAT	G D K
sp:P10639:THIO_MOUSE	G D K
tr:D3DMZ9:D3DMZ9_DROME	S G N
sp:P47938:THIO1_DROME	D D Q
sp:Q9V429:THIO2_DROME	S G N
tr:Q9VUG9:Q9VUG9_DROME	T N N
sp:Q8IFW4:THIOT_DROME	E D G
tr:C8VV89:C8VV89_DROME	E D G
tr:Q9TXY8:Q9TXY8_CAEEL	K T D
sp:P22803:TRX2_YEAST	G D G
sp:P25372:TRX3_YEAST	N D D

	whole protein		domain-based		contact-based	
protein	ident	simil	ident	simil	ident	simil
PDB:3e3e:chainA:P10599	0.98	0.99	0.98	0.99	1.0	1.0
tr:H9ZYJ2:H9ZYJ2_HUMAN	1.0	1.0	1.0	1.0	1.0	1.0
sp:P10599:THIO_HUMAN	1.0	1.0	1.0	1.0	1.0	1.0
tr:R4GNK3:R4GNK3_RAT	0.82	0.93	0.83	0.94	1.0	1.0
sp:P10639:THIO_MOUSE	0.88	0.96	0.88	0.96	1.0	1.0
tr:D3DMZ9:D3DMZ9_DROME	0.49	0.81	0.49	0.81	0.0	0.56
sp:P47938:THIO1_DROME	0.35	0.78	0.36	0.8	0.33	0.56
sp:Q9V429:THIO2_DROME	0.43	0.75	0.43	0.75	0.0	0.56
tr:Q9VUG9:Q9VUG9_DROME	0.3	0.6	0.38	0.78	0.0	0.56
sp:Q8IFW4:THIOT_DROME	0.28	0.53	0.41	0.78	0.33	0.41
tr:C8VV89:C8VV89_DROME	0.26	0.49	0.41	0.78	0.33	0.41
tr:Q9TXY8:Q9TXY8_CAEEL	0.35	0.75	0.35	0.76	0.0	0.41
sp:P22803:TRX2_YEAST	0.48	0.81	0.47	0.8	0.67	0.41
sp:P25372:TRX3_YEAST	0.36	0.63	0.45	0.79	0.33	0.53

dhd (FBgn0011761) associated phenotypes
maternal effect

(Information from FlyBase)

dhd (UniProt:P47938) annotation
Function: Participates in various redox reactions through the reversible oxidation of its active center dithiol to a disulfide and catalyzes dithiol-disulfide exchange reactions. As a reducing substrate of peroxiredoxin 1, thioredoxin 2 is preferred over thioredoxin 1. Required for female meiosis and early embryonic development. (PubMed:11877442, PubMed:9106167).
Subcellular location: Nucleus (PubMed:14579129).
Tissue specificity: Ovary specific. Expressed present in the nurse cells from stage 9 of ovary development and is transported into the oocyte. Expressed throughout oogenesis. (PubMed:14579129).

Developmental stage: Expressed both maternally and zygotically. (PubMed:9106167).
Miscellaneous: The TrxT gene, which encodes an testis specific thioredoxin, is adjacent to the dhd gene and shares some regulatory region with it.

(Information from UniProt)

Trx-2 (FBgn0040070) associated phenotypes

chemical resistant, chemical sensitive, conditional, immune response defective, locomotor behavior defective, long lived, male fertile, oxidative stress response defective, short lived

(Information from FlyBase)

Trx-2 (UniProt:Q9V429) annotation

Function: Participates in various redox reactions through the reversible oxidation of its active center dithiol to a disulfide and catalyzes dithiol-disulfide exchange reactions. As a reducing substrate of peroxiredoxin 1, thioredoxin 2 is preferred over thioredoxin 1. (PubMed:11877442).
Subunit: Monomer. (PubMed:15644209).
Developmental stage: Larval stages. ECO:0000269—PubMed:11877442.

(Information from UniProt)

TrxT (FBgn0029752) associated phenotypes

chemical resistant, long lived

(Information from FlyBase)

TrxT (UniProt:Q8IFW4) annotation

Function: Probably participates in various redox reactions through the reversible oxidation of its active center dithiol to a disulfide and catalyzes dithiol-disulfide exchange reactions. Its tissue specificity suggests a regulatory role in the germline.
Subcellular location: Nucleus (PubMed:14579129). Chromosome (PubMed:14579129). Note=Specifically associated with the Y chromosome loops.
Tissue specificity: Testis specific. Not expressed in the embryo. Becomes progressively more strongly expressed during larval and pupal development. In testis, it is strongly expressed in young spermatocytes, and postmeiotic spermatid stages, then expression decreases at the nuclear elongation stage. Strongly expressed in the waste bag, in which material no longer needed for the mature sperm is eliminated. Not expressed in the stem cells and spermatogonial cells. (PubMed:14579129).
Developmental stage: Not expressed maternally. (PubMed:14579129).
Miscellaneous: The TrxT gene, which encodes an testis specific thioredoxin, is adjacent to the dhd gene and shares some regulatory region with it.

(Information from UniProt)

14-3-3 protein zeta/delta

Best gene implication in ageing for this target family came from gene Q95ZT1 via mapping the annotation from WormBase WBGene00001502 annotated in WormBase 2014-03-11. Annotation GO 8340 (determination of adult lifespan) was Inferred from Mutant Phenotype

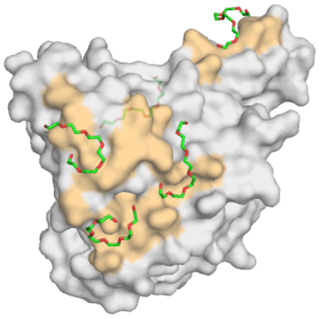

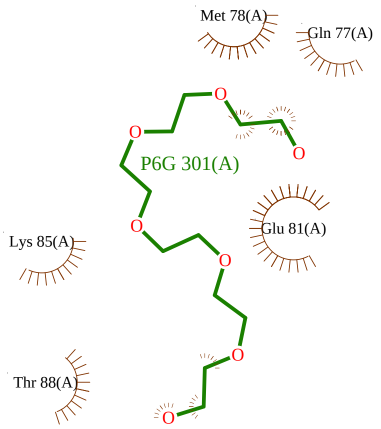

protein	amino acids contacts (binding site)
PDB:4n7g:chainA:P63104	E Y Q G A N A S S Q M E K T S E K P N S A K L K Q A E I E
tr:E7EX29:E7EX29_HUMAN	E Y Q G A N A S S Q M E K T S E K P N S A K L K Q A E I E
sp:P63104:1433Z_HUMAN	E Y Q G A N A S S Q M E K T S E K P N S A K L K Q A E I E
tr:D0PNI1:D0PNI1_HUMAN	E Y Q G A N A S S Q M E K T S E K P N S A K L K Q A E I E
sp:P63102:1433Z_RAT	E Y Q G A N A S S Q M E K T S E K P N S P K L K Q A E I E
sp:P63101:1433Z_MOUSE	E Y Q G A N A S S Q M E K T S E K P N S P K L K Q A E I E
sp:P29310:1433Z_DROME	E Y T G V N A S S Q L E R K G D K P K S P K L K D A D I K
tr:Q95ZT1:Q95ZT1_CAEEL	E Y L G A N A S S Q M E K K N D K P K G A K L K K S E I K
sp:Q20655:14332_CAEEL	E Y L G A N A S S Q M E K K N D K P K G A K L K K S E I K

	whole protein		domain-based		contact-based	
protein	ident	simil	ident	simil	ident	simil
PDB:4n7g:chainA:P63104	1.0	1.0	1.0	1.0	1.0	1.0
tr:E7EX29:E7EX29_HUMAN	0.94	0.94	0.97	0.97	1.0	1.0
sp:P63104:1433Z_HUMAN	1.0	1.0	1.0	1.0	1.0	1.0
tr:D0PNI1:D0PNI1_HUMAN	1.0	1.0	1.0	1.0	1.0	1.0
sp:P63102:1433Z_RAT	1.0	1.0	1.0	1.0	0.97	0.99
sp:P63101:1433Z_MOUSE	1.0	1.0	1.0	1.0	0.97	0.99
sp:P29310:1433Z_DROME	0.81	0.94	0.82	0.95	0.59	0.8
tr:Q95ZT1:Q95ZT1_CAEEL	0.64	0.75	0.69	0.8	0.69	0.79
sp:Q20655:14332_CAEEL	0.78	0.91	0.83	0.95	0.69	0.79

14-3-3zeta (FBgn0004907) associated phenotypes

learning defective, maternal effect, memory defective, mitotic cell cycle defective, neurophysiology defective

(Information from FlyBase)

14-3-3zeta (UniProt:P29310) annotation

Function: Required in Raf-dependent cell proliferation and photoreceptor differentiation during eye development. Acts upstream of Raf and downstream of Ras, and is essential for viability. Acts as a negative regulator by decreasing its voltage sensitivity. Inhibits yki activity by restricting its nuclear localization. (PubMed:10230800, PubMed:19900439).

Subunit: Homodimer; homodimerization is not essential for modulating the activity of Slo. Interacts with phosphorylated Slob; the interaction with Slob mediates an indirect interaction with Slo. Interacts with phosphorylated yki. (PubMed:10230800, PubMed:18256197, PubMed:19900439).

Subcellular location: Cytoplasm ECO:0000250.

Tissue specificity: Predominantly expressed in the ventral nerve cord of the embryo, and in the neural tissues of the head. Also found in the region posterior to the morphogenetic furrow of the eye imaginal disk where cells differentiate as photoreceptors.

Developmental stage: Expressed throughout all stages of embryonic and larval development.

(Information from UniProt)

ftt-2 (WBGene00001502) associated phenotypes
lethal, sterile

(Information from WormBase)

ftt-2 (UniProt:Q20655) annotation
Function: Required for extension of life-span by sir-2.1 (PubMed:16777605). Promotes nuclear export of yap-1 (PubMed:23396260). (PubMed:16777605, PubMed:23396260).
Subunit: Interacts with daf-16 and sir-2.1. (PubMed:16777605).
Subcellular location: Cytoplasm ECO:0000269—PubMed:16777605. Nucleus (PubMed:16777605).

(Information from UniProt)

Peptidyl-prolyl cis-trans isomerase NIMA-interacting 1

Best gene implication in ageing for this target family came from gene Q13526 via mapping the annotation from Ensembl ENSG00000127445 via mapping the annotation from EntrezGene 5300 via mapping the annotation from GenAgeHuman 0062 annotated in GenAge release 17.

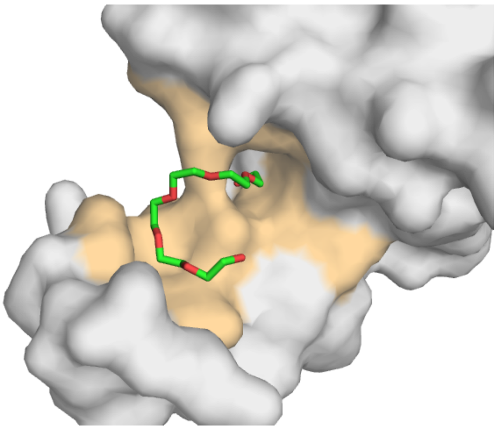

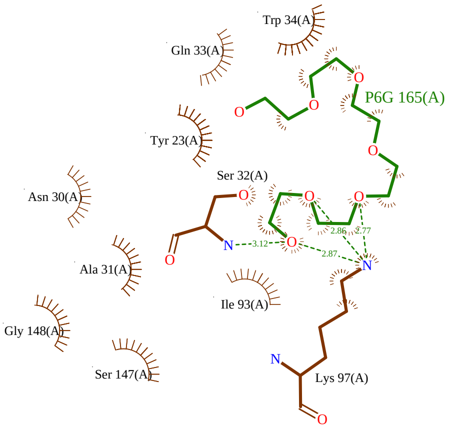

protein	amino acids contacts (binding site)
PDB:3tc5:chainA:Q13526	Y N A S Q W I K S G
sp:Q13526:PIN1_HUMAN	Y N A S Q W I K S G
tr:B0BNL2:B0BNL2_RAT	Y N A S Q W I K S G
tr:Q3ULQ2:Q3ULQ2_MOUSE	Y N A S Q W I K S G
sp:Q9QUR7:PIN1_MOUSE	Y N A S Q W I K S G
sp:P54353:DOD_DROME	Y K E S Q W R V S G
tr:Q9N492:Q9N492_CAEEL	Y G R S Q W E K S D
sp:P22696:ESS1_YEAST	Y K H S Q W I D S D

	whole protein		domain-based		contact-based	
protein	ident	simil	ident	simil	ident	simil
PDB:3tc5:chainA:Q13526	0.99	1.0	0.99	1.0	1.0	1.0
sp:Q13526:PIN1_HUMAN	1.0	1.0	1.0	1.0	1.0	1.0
tr:B0BNL2:B0BNL2_RAT	0.96	0.98	0.99	1.0	1.0	1.0
tr:Q3ULQ2:Q3ULQ2_MOUSE	0.92	0.96	0.95	0.98	1.0	1.0
sp:Q9QUR7:PIN1_MOUSE	0.95	0.98	0.98	1.0	1.0	1.0
sp:P54353:DOD_DROME	0.55	0.82	0.58	0.84	0.6	0.63
tr:Q9N492:Q9N492_CAEEL	0.54	0.81	0.57	0.84	0.6	0.61
sp:P22696:ESS1_YEAST	0.44	0.78	0.46	0.8	0.6	0.65

NAD-dependent protein deacetylase sirtuin-2

Best gene implication in ageing for this target family came from gene Q9I7I7 annotated in UniProt release 2014_02. Annotation GO 8340 (determination of adult lifespan) was Inferred from Mutant

Phenotype

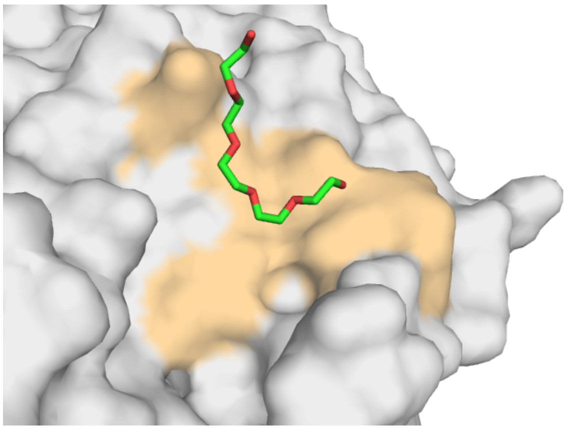

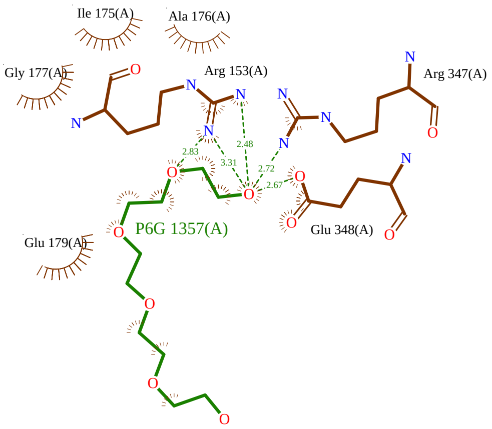

protein	amino acids contacts (binding site)						
PDB:3zgo:chainA:Q8IXJ6	R	I	A	G	E	R	E
sp:Q8IXJ6:SIR2_HUMAN	R	I	A	G	E	R	E
sp:Q5RJQ4:SIR2_RAT	R	V	A	G	E	R	E
tr:Q3UJK6:Q3UJK6_MOUSE	R	V	A	G	E	R	E
sp:Q8VDQ8:SIR2_MOUSE	R	V	A	G	E	R	E
sp:Q9I7I7:SIRT2_DROME	R	L	T	G	P	S	E
sp:P53686:HST2_YEAST	K	Q	A	G	K	A	Q

	whole protein		domain-based		contact-based	
protein	ident	simil	ident	simil	ident	simil
PDB:3zgo:chainA:Q8IXJ6	0.99	1.0	1.0	1.0	1.0	1.0
sp:Q8IXJ6:SIR2_HUMAN	1.0	1.0	1.0	1.0	1.0	1.0
sp:Q5RJQ4:SIR2_RAT	0.78	0.87	0.89	0.97	0.86	0.95
tr:Q3UJK6:Q3UJK6_MOUSE	0.79	0.87	0.89	0.97	0.86	0.95
sp:Q8VDQ8:SIR2_MOUSE	0.88	0.97	0.89	0.97	0.86	0.95
sp:Q9I7I7:SIRT2_DROME	0.43	0.72	0.54	0.83	0.43	0.6
sp:P53686:HST2_YEAST	0.29	0.57	0.38	0.68	0.29	0.57

Sirt2 (FBgn0038788) associated phenotypes

heat sensitive, lethal - all die before end of pupal stage, some die during pupal stage

(Information from FlyBase)

Sirt2 (UniProt:Q9I7I7) annotation

Function: NAD-dependent protein deacetylase (By similarity). May be involved in the regulation of life span. (, PubMed:17159295).

Cofactor: Zn(2+)Note=Binds 1 zinc ion per subunit. ;

Disruption phenotype: Causes lethality during development. Induced silencing shortens life span. (PubMed:17159295).

(Information from UniProt)
